# Linac-based radiotherapy for epicondylitis humeri

**DOI:** 10.17179/excli2020-1069

**Published:** 2020-03-04

**Authors:** Mahmoud Navaser, Hamed Ghaffari, Mehrnaz Mashoufi, Soheila Refahi

**Affiliations:** 1 Department of Medical Physics, School of Medicine, Iran University of Medical Sciences, Tehran, Iran; 2 Department of Health Information Management, Faculty of Medicine, Ardabil University of Medical Sciences, Ardabil, Iran; 3 Department of Medical Physics, Faculty of Medicine, Ardabil University of Medical Sciences, Ardabil, Iran

## ⁯⁯

***Dear Editor,***

Radiotherapy is considered as one of the most effective treatment options for epicondylitis humeri. In the past, orthovoltage machines were used to treat humeral epicondylitis. Within the past few years, owing to technological improvement, an increasing shift to linac-based radiotherapy for humeral epicondylitis has taken place. This letter highlights the role of linac-based radiotherapy for epicondylitis humeri, and also discusses considerations and perspectives. 

Humeral epicondylitis is considered as a relatively common disorder of the arm. Depending on the etiology of disorder, it is classified into (i.) the lateral epicondylitis, termed as tennis elbow; (ii.) the medial epicondylitis, termed as golfer's elbow (Hauptmann et al., 2020[3], 2019[4]; Leszek et al., 2015[7]). Lateral epicondylitis is more frequent than the medial epicondylitis (Hauptmann et al., 2020[3]). Humeral epicondylitis has a negative impact on patients' quality of life. The most important symptoms are elbow pain, joint mobility restriction, and local tenderness. Also, in some cases, increased local temperature and slight joint edema have been seen. Repetitive movement at first glance and extensive computer work are main risk factors for humeral epicondylitis. 

In spite of availability of several therapeutic options for treating epicondylitis humeri, radiotherapy remains a conservative treatment modality that is widely used in Western Europe. There is a long history and robust theoretical background supporting effectiveness of radiotherapy for patients with humeral epicondylitis. During the last 30 years, a number of studies have been conducted regarding radiotherapy in patients with tennis/golfer's elbow (Hauptmann et al., 2019[4]). 

Data of published studies show that the vast majority of patients were treated with orthovoltage therapy and some others were irradiated with Cobalt and Caesium device (Hauptmann et al., 2019[4]). Within the past few years, technological revolution led to substantially changing technical equipment. For example, nowadays, there is no institution to treat patients with cobalt units in Germany. In addition, due to the decreasing availability of orthovoltage machines and lack of appropriate substitute devices, institutions do not have the opportunity to utilize orthovoltage therapy. Also, most of the orthovoltage machines are old (Kriz et al., 2018[6]). Therefore, an increasing shift to linac-based radiotherapy for humeral epicondylitis has taken place. To the best of our knowledge, there are only four published studies reporting linac-based radiotherapy for epicondylitis humeri (Hauptmann et al., 2020[3], 2019[4]; Leszek et al., 2015[7]; Schlehuber, 2004[11]).

### Clinical outcomes

Before 2018, there were only two published studies with small sample assessing the effectiveness of linac-based radiotherapy in patients with epicondylitis humeri (Leszek et al., 2015[7]; Schlehuber, 2004[11]). In the first study, 34 cases underwent radiotherapy (Schlehuber, 2004[11]). The response rate, complete response rate, and partial response rate were 27 %, 1 %, and 26 %, respectively. In another study, Leszek et al. reported that 50 patients treated with a linear accelerator for epicondylitis humeri and the response rate, complete response rate, and partial response rate were 70 %, 30 %, and 40 %, respectively (Leszek et al., 2015[7]).

More recently, two retrospective studies from the University of Regensburg have investigated the effectiveness of radiotherapy with a linear accelerator for humeral epicondylitis, and also reported the results of a re-irradiation for these patients (Hauptmann et al., 2020[3], 2019[4]). In 2019, Hautmann et al. have reported the results of radiotherapy with a 6MV linear accelerator for treating 124 patients (138 elbows) (Hauptmann et al., 2019[4]). It was the first large study on treating humeral epicondylitis with a linac. The vast majority of elbows were treated with a fractionated dose of 1.0 Gy to a total dose of 6.0 Gy, and in some others, 0.5 Gy to a total dose of 3.0 Gy. The response rate, complete response rate, and partial response rate were 70 %, 64 %, and 6 %, respectively. The median pain was 7 according to the numeric rating scale (NRS) prior to radiotherapy, 4 after 6 weeks and 0 after 12 and 24 months. There was a significant pain reduction compared with the pain level before radiotherapy during the entire follow-up time (*P < 0.0001*) (Hauptmann et al., 2019[4]). 

In 2020, a study by the same group reported the first systematically examining re-irradiation for 99 elbows with epicondylitis humeri (Hauptmann et al., 2020[3]). The median pain score was 6 on the NRS before re-irradiation, 3 after 6 weeks, 2 after 12 months and 1 after 24 months. Data showed that 50.9 % of patients 24 months after re-irradiation were free of pain or with very little pain. For the entire follow-up, there was a statistically significant pain reduction compared with the pain level before re-irradiation (*P < 0.0001*) (Hauptmann et al., 2020[3]).

### Is linac-based radiotherapy superior to orthovoltage?

Linac-based radiotherapy of epicondylitis humeri has demonstrated the beneficial in the previous studies (Hauptmann et al., 2020[3], 2019[4]; Leszek et al., 2015[7]; Schlehuber, 2004[11]). Nowadays, radiotherapy of epicondylitis humeri with a linear accelerator has the widespread use and is an effective and safe treatment modality. However, a direct comparison between linac-based radiotherapy and orthovoltage therapy in terms of clinical outcomes is difficult. Firstly, there is no study comparing effectiveness of orthovoltage therapy and linac-based radiotherapy for epicondylitis humeri. Secondly, from a physicist point of view, there are several differences between orthovoltage machines and linear accelerators such as dose rate, depth dose, energy, etc. Besides, the inclusion criteria, response criteria, and follow-up period are different among studies. 

Nevertheless, a previous study by Hautmann et al. using a case-related analysis has indicated that the results of published studies using orthovoltage therapy for epicondylitis humeri since 1990 outperform photon- or gamma-based radiotherapy in terms of the overall response rate and the partial response rate (Hauptmann et al., 2019[4]). However, there was no significant difference in the complete response rate between two treatment modalities. According to published studies, it should be noted that approximately 12 % (321 of 2714 elbows) of the elbows were treated with linac-based radiotherapy for initial irradiation or re-irradiation (Hauptmann et al., 2020[3], 2019[4]). A prospective randomized study will be required to compare the effectiveness of linac-based radiotherapy and orthovoltage therapy.

### Re-irradiation for epicondylitis humeri

The reasons for re-irradiation are including no response or partial response to initial radiation and recurrent pain. Therefore, a second or third radiotherapy course was performed to achieve desired outcome. Of note, in some institutions, two courses of radiotherapy with an interval of 6 weeks are considered as a primary treatment (Seegenschmiedt et al., 1997[13]; Seegenschmiedt and Keilholz, 1998[12]). A previous study showed that the median re-irradiation was 12 weeks with a range 6 weeks to 119 months (Hauptmann et al., 2020[3]). Re-irradiation with a linear accelerator results in a significant reduction of pain regardless of the reason for re-irradiation (Hauptmann et al., 2020[3]). Two previous studies with orthovoltage machines applied third course of irradiation for epicondylitis humeri (Reinhold and Sauerbrey, 1961[10]; Ketterer, 2007[5]). However, there is a good response to the second re-irradiation (Hauptmann et al., 2020[3]). Therefore, in lack of sufficient data, a third course of radiotherapy can be ethical. Although, the previous study has indicated that re-irradiation for epicondylitis humeri with a linear accelerator is an effective and safe method (Hauptmann et al., 2020[3]), a general application of two or more series of radiotherapy is not recommended owing to radiation protection reasons and minimization of potential radiation risks. Randomized trials are needed to elucidate the effect of re-irradiation with a linear accelerator on humeral epicondylitis, as well as risk-benefit ratio; however, it should be noted that performing controlled randomized trials for re-irradiation is very challenging because it is given to a specific selected sample.

### Dose concept and filed arrangement

There is no randomized study and, therefore, definitive recommendations regarding the exact dose concept for radiotherapy of humeral epicondylitis remain nebulous. The radiation doses and fractionation schemes that are currently used in the routine clinical practice have been determined empirically. Nevertheless, previous studies have reported that there is no significant difference in pain reduction between patients treated with 6 times 0.5 Gy compared to those with 6 times 1.0 Gy (Hauptmann et al., 2020[3], 2019[4]). These results are in good agreement with a previous study by Ott et al. who demonstrated there is no significant difference in in the quality of the long-term response between two dose fractionation schedules (i.e., 0.5 Gy or 1.0 Gy) (Ott et al., 2014[8]). Also, previous study suggests that the effect of low dose radiotherapy is maintained up to 48 h after treatment and lost at 72 h (Arenas et al., 2012[1]). In addition, low dose radiotherapy, especially a single dose per fraction of 0.5 Gy has no harmful effect (Deloch et al., 2018[2]). Taken together, for reasons of radiation protection a single dose of 0.5 Gy and a total dose of 3.0 Gy over 2-3 weeks using 6 MV photons in opposing fields seems to be recommendable for initial irradiation. A single dose of 1.0 Gy to a total dose of 6.0 Gy using 6 MV photons in opposing fields can be prescribed for re-irradiation. Depending on the results of the clinical examination, the fields are defined by an experienced physician. Using bolus material may be required in some cases.

### Radiobiological effect and other considerations

Despite the widespread use of radiotherapy for epicondylitis humeri, the radiobiological mechanisms involved in the anti-inflammatory effects of low dose radiotherapy have not been completely elucidated. However, it is worthwhile to mention that mechanisms underlying the anti-inflammatory effects of low dose radiotherapy involve following potential mediators: reduced expression of L-, E-selectins, a decrease in nitric oxide (NO), an increase in apoptosis, increased activation of nuclear factor-kappa B (NF-κB), and an increase in the expression of anti-inflammatory cytokines such as transforming growth factor β1 (TGF-β1) and interleukin-10 (IL-10) (Arenas et al., 2012[1]). 

There is no clear information about target volumes in published studies. To delineate an exact target volume computed tomography (CT) data and magnetic resonance imaging (MRI) data are required. It is recommended that the target volume should encompass the complete epicondyle (lateral or medial) together with the nearby bony and muscular tissues (Ott et al., 2015[9]). To date, radiotherapy has been used to treat epicondylitis humeri in the large number of patients, demonstrating the wide acceptance of this treatment option. For example, studies show that in Germany, approximately 3500 patients with epicondylitis humeri receive radiotherapy annually (Hauptmann et al., 2020[3], 2019[4]). Therefore, it is necessary to manage the workload imposed on the linac, in-room time and personnel involved in these departments. It should be noted, however, that the main focus of these departments is on cancer treatment. In addition, training of radiation technicians and continuous education of physicians is needed to improved treatment concept and interdisciplinary cooperation. It is necessary to improve treatment guidelines for radiotherapy and develop technical and clinical quality assurance criteria. Randomized controlled multicenter trials will be required to clarify effectiveness of linac-based radiotherapy and optimize treatment schedules (*i.e.*, prescription dose, fractionation scheme, and treatment time). Such a study will encourage other countries to use radiotherapy for epicondylitis humeri. Radiotherapists may ignore linac-based radiotherapy for epicondylitis humeri because its reimbursement is low. Thus, changes in reimbursement are required. 

## Funding

Not applicable.

## Conflict of interests

The authors declare that they have no conflict of interest.

## Research involving human participants and/or animals

This article does not contain any studies with human participants or animals performed by any of the authors.

## Informed consent

Consent is not required for this type of study.
